# Minimum inhibitory concentrations of azithromycin in clinical isolates of *Mycobacterium avium* complex in Japan

**DOI:** 10.1128/spectrum.00218-24

**Published:** 2024-04-30

**Authors:** Yoshifumi Uwamino, Wataru Aoki, Rika Inose, Yuka Kamoshita, Kei Mikita, Ho Namkoong, Tomoyasu Nishimura, Hiromichi Matsushita, Naoki Hasegawa

**Affiliations:** 1 Department of Laboratory Medicine, Keio University School of Medicine, Tokyo, Japan; 2 Department of Infectious Diseases, Keio University School of Medicine, Tokyo, Japan; 3 Clinical Laboratory, Keio University Hospital, Tokyo, Japan; 4 Keio University Health Center, Tokyo, Japan; Johns Hopkins University School of Medicine, Baltimore, Maryland, USA

**Keywords:** *Mycobacterium avium *complex, azithromycin, clarithromycin, minimum inhibitory concentration

## Abstract

The latest guidelines include azithromycin as a preferred regimen for treating *Mycobacterium avium* complex (MAC) pulmonary disease. However, serially collected susceptibility data on clinical MAC isolates are limited, and no breakpoints have been determined. We investigated the minimum inhibitory concentrations (MICs) of azithromycin and clarithromycin for all MAC strains isolated in 2021 from a single center in Japan, excluding duplicates. The MICs were determined using a panel based on the microbroth dilution method, according to the latest Clinical and Laboratory Standards Institute recommendations. The MICs were determined for 318 MAC strains. Although there was a significant positive correlation between the MICs of azithromycin and clarithromycin, the MICs of azithromycin tended to be higher than those of clarithromycin. Among the cases in which the strains were isolated, 18 patients initiated treatment, including azithromycin treatment, after sample collection. Some patients infected with stains with relatively high azithromycin MICs achieved a microbiological cure with azithromycin-containing regimens. This study revealed a higher MIC distribution for azithromycin than clarithromycin, raising questions about the current practice of estimating azithromycin susceptibility based on the clarithromycin susceptibility test result. However, this was a single-center study that included only a limited number of cases treated with azithromycin. Therefore, further multicenter studies that include a greater number of cases treated with azithromycin are warranted to verify the distribution of azithromycin MICs and examine the correlation between azithromycin MICs and treatment effectiveness.

**IMPORTANCE:**

The macrolides serve as key drugs in the treatment of pulmonary *Mycobacterium avium* complex infection, and the administration of macrolide should be guided by susceptibility test results. Azithromycin is recommended as a preferred choice among macrolides, surpassing clarithromycin; however, drug susceptibility testing is often not conducted, and clarithromycin susceptibility is used as a surrogate. This study represents the first investigation into the minimum inhibitory concentration of azithromycin on a scale of several hundred clinical isolates, revealing an overall tendency for higher minimum inhibitory concentrations compared with clarithromycin. The results raise questions about the appropriateness of using clarithromycin susceptibility test outcomes for determining the administration of azithromycin. This study highlights the need for future discussions on the clinical breakpoints of azithromycin, based on large-scale clinical research correlating azithromycin susceptibility with treatment outcomes.

## INTRODUCTION

The *Mycobacterium avium* complex (MAC) is an environmental bacterium found in soil and water. In humans, it primarily leads to refractory chronic respiratory infections, necessitating long-term combination therapy with multiple drugs (1). Macrolide is a key drug in combination therapy for MAC pulmonary disease according to the susceptibility of the infecting strain. Although clarithromycin (CAM) is generally used for the combination therapy, the American Thoracic Society (ATS)/European Respiratory Society (ERS)/European Society of Clinical Microbiology and Infectious Diseases (ESCMID)/Infectious Diseases Society of America (IDSA) clinical practice guidelines for treatment of nontuberculous mycobacterial pulmonary disease, revised in 2020, recommend using azithromycin (AZM) in preference to CAM because AZM has higher tolerability, fewer drug interactions, lower pill burden, and more convenient dosing than CAM, and the two antibiotics have similar efficacy (2). Additionally, the half-life of AZM is longer than that of CAM, and the pulmonary tissue penetration of AZM is superior to that of CAM, which might contribute to its effectiveness (3).

Based on the results of previous studies that have demonstrated that CAM susceptibility test results are correlated with clinical outcomes (4–6), macrolides and amikacin susceptibility-based treatment are recommended for patients with MAC pulmonary disease. However, AZM susceptibility testing is rarely performed even under the current guideline, which recommends AZM in preference to CAM. AZM-containing regimens are initiated according to the CAM susceptibility results. Currently, limited AZM susceptibility data are available on serially collected clinical MAC isolates, and no breakpoints have been determined.

In Japan, antimicrobial susceptibility testing of MAC isolates was performed primarily using BrothMIC NTM (Kyokuto Pharmaceutical Industrial.,co. Ltd, Tokyo, Japan) susceptibility test panels, which measured the minimum inhibitory concentration (MIC) for CAM but not AZM. Furthermore, the BrothMIC NTM panels used the Middlebrook 7H9 broth-based broth microdilution method based on the Clinical and Laboratory Standards Institute (CLSI) M24A recommendations (7), which differed from the method recommended in the current CLSI (CLSI M24A3) guidelines (8). In April 2023, the cation-adjusted Mueller–Hinton broth (CAMHB)-based broth microdilution method susceptibility test panel BrothMIC SGM (Kyokuto Pharmaceutical Industrial. co. Ltd, Tokyo, Japan) for slowly growing mycobacteria became available, which can measure the MIC of both CAM and AZM according to the CLSI M42A3 recommendation. Therefore, we performed AZM susceptibility surveillance using serially collected clinical MAC isolates to describe the epidemiology of AZM resistance and determine its relationship to CAM resistance and macrolide resistance mutations.

## MATERIALS AND METHODS

### Patients and samples

This retrospective cross-sectional study included all patients with MAC-positive mycobacterial cultures collected between January and December 2021 at Keio University Hospital (Tokyo, Japan). Keio University Hospital is situated in the center of Tokyo and a referral center for non-tuberculosis mycobacterial (NTM) disease in Japan, treating approximately 700 patients with NTM annually. During the study period, the samples for mycobacterial culture were incubated in an in-house microbiology laboratory using mycobacteria growth indicator tubes (MGIT) and BD MGIT 960 incubators (Beckton and Dickenson, Franklin Lakes, NJ, USA) for 8 weeks, and the isolates in positive tubes were cultured on Ogawa medium and identified using mass spectrometry (Bruker Biotyper, Bruker, MA, USA). Antimicrobial susceptibility testing was performed using BrothMIC NTM panels according to the manufacturer’s recommendations. All isolates were stored in MGIT tubes. In this study, the stored MAC isolates from all included patients were analyzed. If the patient had more than one MAC isolate cultured during the study period, the isolate from the earliest positive sample collected during the period was included in the analysis. If different MAC strains were isolated from a single positive sample, each strain was included in the analysis.

Patient medical charts were retrospectively reviewed to extract information on their age, sex, and diagnosis. Patients’ age, sex, and whether they were diagnosed with pulmonary MAC infection in accordance with the ATS/ERS/ESCMID/IDSA guidelines were confirmed using electronic medical records. To determine whether prior exposure to macrolide antibiotics was related to AZM and CAM susceptibility, the history of macrolide antibiotic administration was verified. Short-term administration of macrolide antibiotics for conditions, such as acute pneumonia was not included in the administration history.

Furthermore, we identified untreated patients (those without a history of antimicrobial therapy for nontuberculous mycobacterial infections or prolonged monotherapy with macrolides) at the time of collecting MAC-positive samples. Among the untreated patients, we investigated the posttreatment outcomes of those who initiated therapy for pulmonary MAC infection with two or more antimicrobial agents, including AZM, after the collection of MAC-positive samples. Culture conversion and treatment failure were assessed according to the Nontuberculous Mycobacterial Network (NTM-NET) consensus statement (9).

The study protocol was approved by the Ethics Committee of the Keio University School of Medicine (20231014). The requirement for written informed consent was waived because of the retrospective observational nature of the study.

### Broth microdilution testing

Broth microdilution tests were performed using BrothMIC SGM test panels to determine the MICs of AZM and CAM. BrothMIC SGM test panels are broth microdilution method-based panels with cation-adjusted Mueller–Hinton broth containing 5% oleic albumin dextrose catalase (OADC), under the conditions described in the CLSI M24A3 recommendations. Serial dilutions of antibiotic were added to each well to measure the minimum concentration at which growth was inhibited. The final antibiotic concentrations were 0.06–64 µg/mL for CAM, 0.06–64 µg/mL for AZM, 0.25–8 µg/mL for moxifloxacin, 0.125–4 µg/mL for sitafloxacin, 8–256 µg/mL for amikacin, 2–64 µg/mL for kanamycin, 0.5–16 µg/mL for minocycline, 0.5–16 µg/mL for doxycycline, 0.125–4 µg/mL for isoniazid, 2–64 µg/mL for linezolid, 0.5–16 µg/mL for ethambutol, 0.5–16 µg/mL for ethionamide, 0.25–8 µg/mL for rifabutin, and 0.125–4 µg/mL for rifampicin.

Stored MAC isolates were inoculated into Ogawa medium and incubated for approximately 2 weeks. A single fresh colony on the medium was dissolved in MycoBroth (Kyokuto Pharmaceutical Industrial.,co. Ltd, Tokyo, Japan), incubated for 3 days, and adjusted to a McFarland turbidity of 0.5 using a Vi-Spec II turbidity meter (Kyokuto Pharmaceutical Industrial. co. Ltd, Tokyo, Japan). Then, 55 µL of the bacterial solution was mixed with 11 mL of sterile purified water, and 100 µL of the adjusted mixture was inoculated into each well of the BrothMIC SGM panel. The panels were incubated at 36°C for 7 days to confirm growth in the control wells. If no growth was observed in the control well after 7 days, it was reassessed on day 14, and the MIC for each antimicrobial agent was evaluated for those in which growth was observed.

The MIC was evaluated blindly by three laboratory technicians, and if two or more laboratory technicians agreed, that value was determined as the MIC value. If all three technicians' judgments differed, the isolate was excluded from the analysis based on “discrepant results.” Quality control of MIC measurements was performed using *Mycobacteirum marinum* ATCC927 according to the conditions specified in CLSI M24A3.

### Detection of macrolide-resistant mutations

Macrolide resistance in MAC is generally explained by a point mutation at position 2,058 or 2,059 of the 23S rRNA gene (*rrl* gene mutation). To evaluate the relationship between *rrl* gene mutations and AZM MICs, *rrl* gene mutations were identified in all stored MAC isolates using amplification refractory mutation system (ARMS)-PCR methods. From stored MAC isolates in MGIT tubes, bacterial DNA was extracted using the Cica Genious DNA Extraction Reagent (Kanto Chemical Co., Inc., Tokyo, Japan). The primers and conditions for ARMS PCR have been described previously (10). Takara Taq Hot start reagents (Takara Bio Inc., Shiga, Japan) and a Miniamp plus MiniAmp Plus Thermal cycler (Thermo Fisher Scientific, Waltham, MA, USA) were used for amplification. Amplicons were subjected to electrophoresis to determine whether a mutation was present in position 2,058 or 2,059, and classified as wild type or mutant.

### Statistical analysis

The distribution of the MIC of all analyzed isolates measured by broth MIC SGM panels were described. Additionally, the values at which 50% and 90% of isolates were inhibited (MIC_50_ and MIC_90_, respectively) were determined. The relationship between the AZM MIC and CAM MIC was analyzed. Furthermore, the CAM MIC and AZM MIC were assessed according to the species, presence of *rrl* mutations, and history of macrolide antibiotic administration. Logarithmically transformed MIC values were used in the analysis. If the MIC of an isolate was higher than the upper limit of the measurement, it was treated as twice the upper limit of the measured MIC (for example, an MIC of >64 µg/mL was treated as 128 µg/mL). A paired *t*-test was performed to compare the logarithms of AZM and CAM MIC distribution. and the Pearson correlation coefficient was calculated to assess their correlation. Furthermore, because they were not normally distributed, Mann–Whitney *U*-tests were performed to assess the significance of differences in the distribution of the logarithms of the AZM and CAM MICs according to the species, presence of *rrl* mutations, and macrolide exposure history. All statistical analyses were performed using JMP Pro 17 software (SAS Institute Inc., Cary, NC, USA).

## RESULTS

### Patient characteristics

During the study period, 318 patients tested positive for MAC. Among them, two patients without stored MAC isolates were excluded from the study. Additionally, samples from patients were mixed with *M. avium* and *Mycobacterium intracellulare*. For samples in which two bacterial species were isolated, each species was treated as a distinct isolate and subjected to individual analysis. Therefore, a total of 318 isolates from 316 patients were included in the analysis. The characteristics of the patients and isolates are shown in Table 1. The patients were predominantly older adults (median age: 72 years) and predominantly (74.4%) female. Sputum was the predominant sample type (94.0%), and 90.2% of the patients had already been diagnosed with MAC pulmonary infection. Approximately half (45.9%) of the patients had a history of macrolide treatment. The predominant bacterial species identified was *M. avium*, accounting for 84.3% of the cases. Only 23 strains harbored *rrl* mutations.

**TABLE 1:** Characteristics of patients and isolates^*b*^

Characteristic	Value
Patients (*N* = 316)	
Age (years), median (IQR)	72 (64–78)
Sex	
Male	81 (25.6%)
Female	235 (74.4%)
Diagnosed with MAC pulmonary infection	285 (90.2%)
Prior history of macrolide treatment	145 (45.9%)
Sample type	
Sputum	299 (94.0%)
Gastric fluid	10 (3.1%)
BALF	10 (3.1%)
AFB smear	
Negative	188 (59.5%)
Indeterminate	54 (17.1%)
1+	39 (12.3%)
2+	25 (7.9%)
3+	10 (3.2%)

Isolates (*N* = 318)	
Species	
*M. avium*	268 (84.3%)
*M. intracellulare*	50 (15.7%)
*rrl* mutation^*a*^	
Wild type	294 (92.7%)
Mutant	23 (7.3%)

^*a*^ One strain that could not determine the presence of *rrl* mutation with repeated ARMS PCR test was eliminated.

^*b*^ AFB, acid-fast bacilli; BALF, bronchoalveolar lavage fluid; IQR, interquartile range; MAC, *Mycobacterium avium* complex.

### Antimicrobial susceptibility testing results

The distribution of the MICs measured by the BrothMIC SGM is shown in Table 2. The AZM MIC results of four isolates and CAM MIC results of eight isolates were excluded from analysis owing to discrepancies in the reading of the results by the three technicians.

**TABLE 2:** Distribution of minimum inhibitory concentration (MIC) according to antibiotic type

Antimicrobial	Analyzed isolates	Discrepant results	Distribution of MIC														MIC_50_	MIC_90_
					>64	64	32	16	8	4	2	1	0.5	0.25	0.125	≤0.06		
Clarithromycin	310	8			28	0	2	4	5	18	67	67	95	20	4	0	1	16
Azithromycin	314	4			34	20	71	84	81	19	5	0	0	0	0	0	16	>64
			>256	256	128	64	32	16	≤8									
Amikacin	307	11	5	6	12	32	52	78	122								16	64
					>64	64	32	16	8	4	≤2							
Kanamycin	300	18			14	32	62	80	82	20	10						16	64
Linezolid	300	18			26	139	54	34	36	7	4						64	64
						>32	32	16	8	4	≤2							
Ethambutol	314	4				170	0	93	41	9	1						>32	>32
						>32	32	16	8	4	2	1	≤0.5					
Ethionamide	297	21				127	0	76	38	32	17	5	2				16	>32
							>16	16	8	4	2	1	≤0.5					
Minocycline	301	17					41	87	77	57	35	2	2				8	>16
Doxycycline	301	17					212	18	28	33	7	1	2				>16	>16
								>8	8	4	2	1	0.5	≤0.25				
Moxifloxacin	308	10						6	12	27	83	70	53	57			1	4
Rifabutin	315	3						4	0	0	4	4	8	295			≤0.25	≤0.25
									>4	4	2	1	0.5	0.25	≤0.125			
Sitafloxacin	300	18							12	12	26	50	82	52	66		0.5	2
Rifampicin	311	7							78	71	56	52	33	13	8		2	>4
									>4	4	2	1	≤0.5					
Isoniazid	317	1							191	76	46	3	1				>4	>4

**Fig 1:**
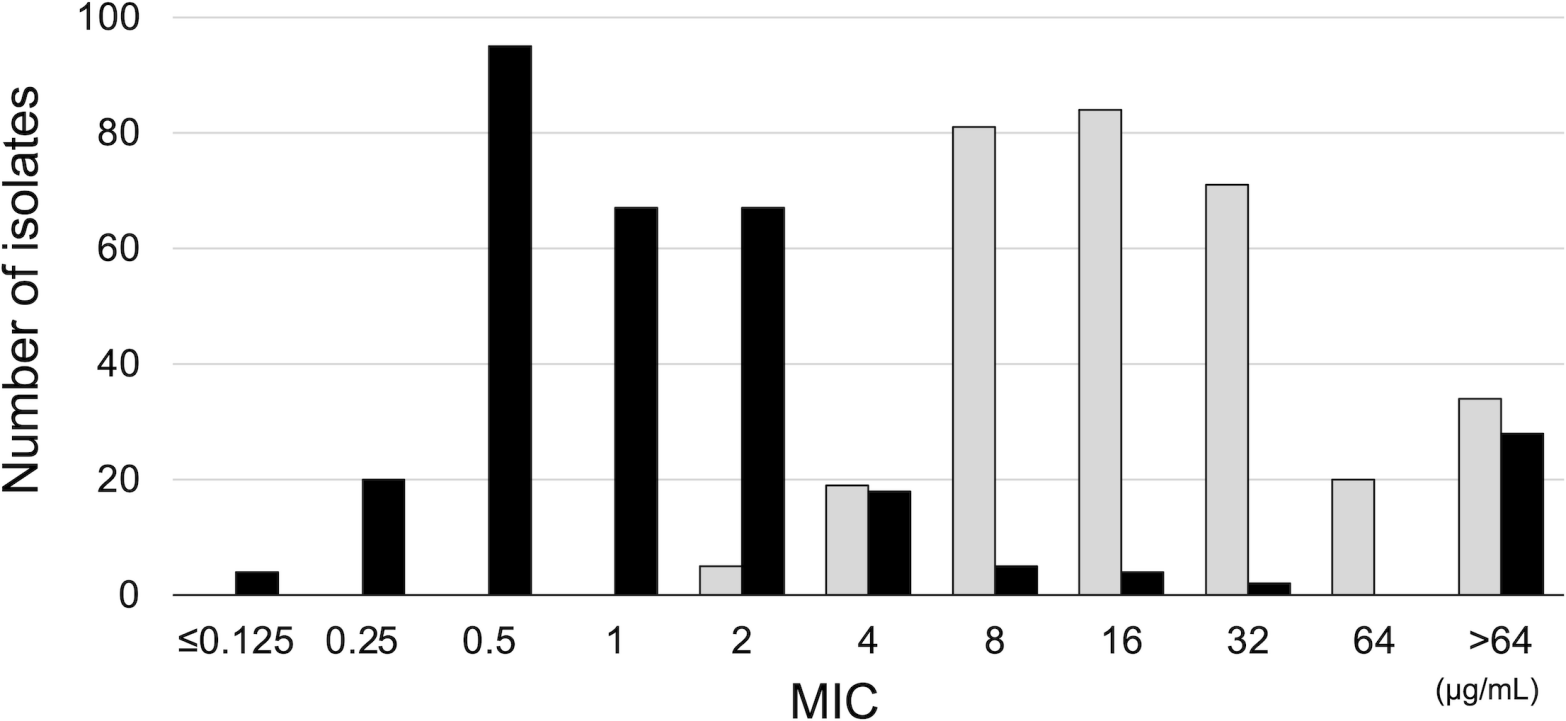
Distribution of clarithromycin and azithromycin MICs. The number of isolates in each clarithromycin MIC category was determined. The black bars represent the clarithromycin MICs, and the gray bars represent the azithromycin MICs.

The distribution of the CAM MIC and AZM MIC are also shown in Fig. 1. The MIC_50_ and MIC_90_ for CAM were 1 and 16 µg/mL, respectively. The MIC_50_ and MIC_90_ for AZM were 16 and >64 µg/mL, respectively. The logarithmically transformed AZM MIC (Log_2_AZM-MIC) values were significantly higher than the logarithmically transformed CAM MIC (Log_2_CAM-MIC) values (*P* < 0.001, paired *t*-test).

**Fig 2:**
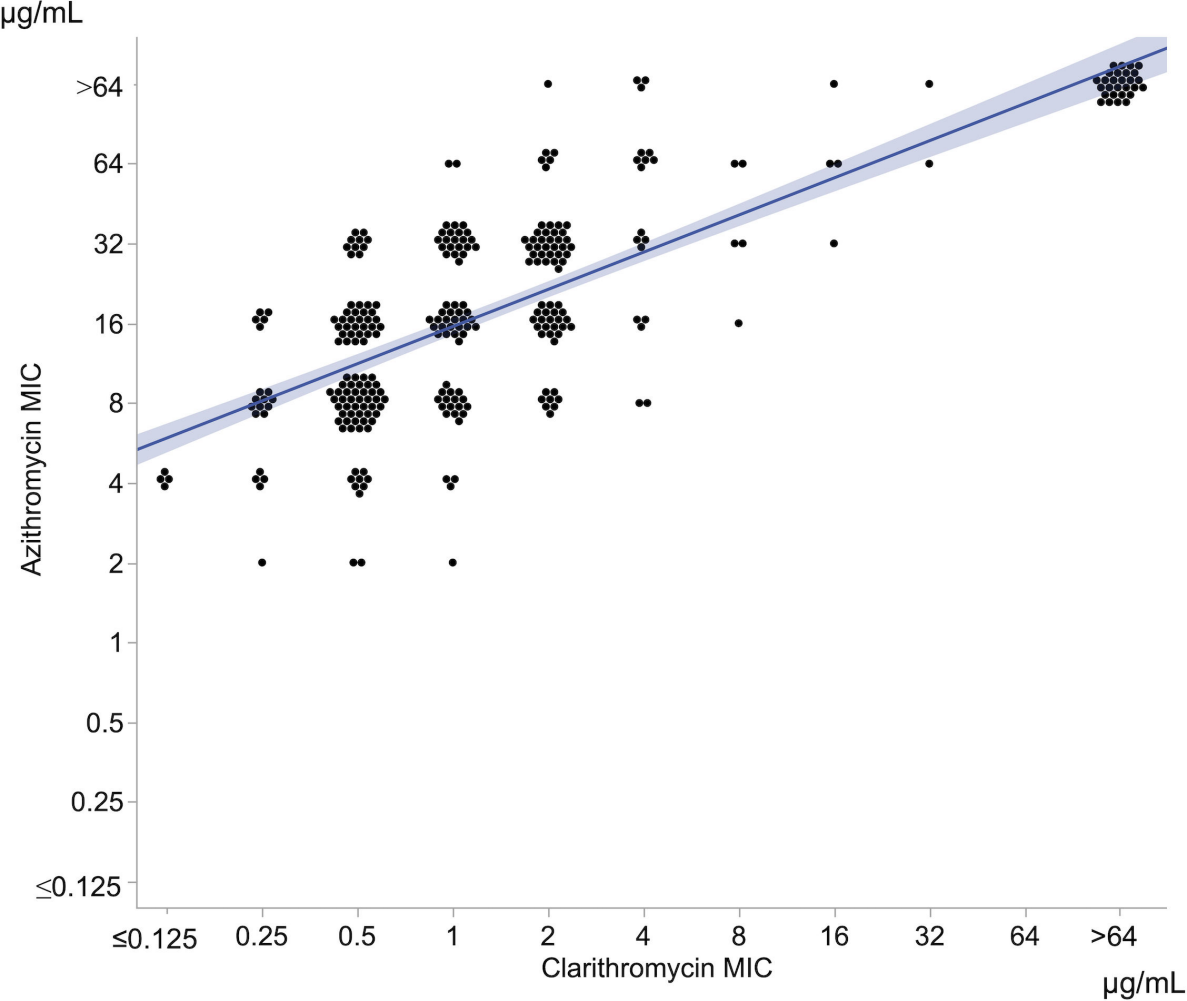
Correlation between the clarithromycin and azithromycin MICs. Scatter dot plots showing the relationship between the clarithromycin and azithromycin MICs. Each dot represents one isolate. The regression line and 95% CI are shown in blue.

Figure 2 shows the relationship between the AZM MIC and CAM MIC for each isolate. The Pearson correlation coefficient of Log_2_AZM-MIC and Log_2_CAM-MIC was 0.784 (95% CI: 0.737–0.824, *P* < 0.001).

### Differences in the AZM and CAM MICs according to the species, presence of *rrl* mutations and patient macrolide exposure

**Fig 3:**
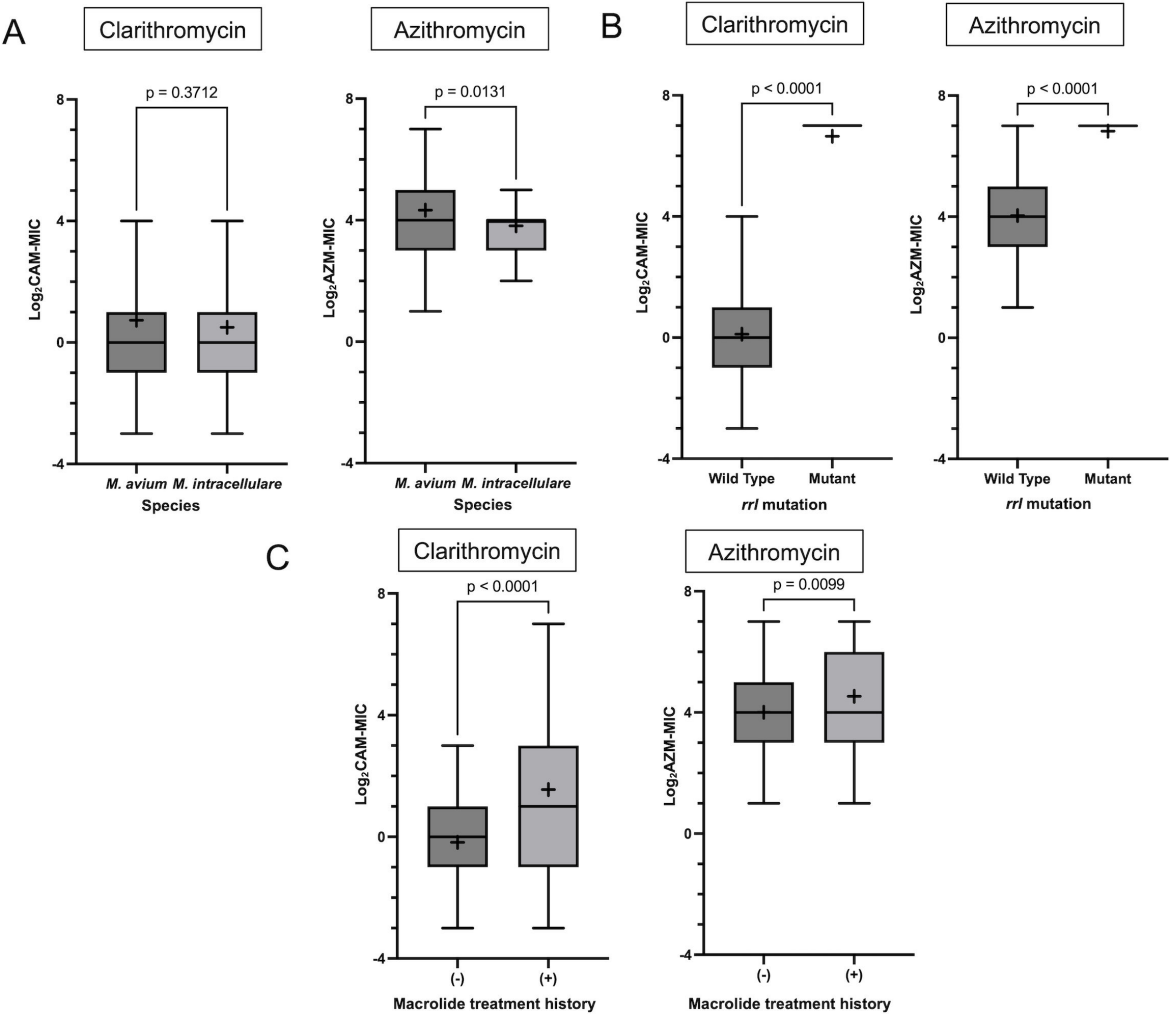
Differences in the MICs according to species, presence of *rrl* mutations, and history of prior macrolide therapy. The bars show the median of the logarithms of CAM and AZM MICs (Log_2_CAM-MIC and Log_2_AZM-MIC), the boxes show the quartile range, and the error bars show the maximum and minimum after exclusion of outliers. “+” signs demonstrate the mean of Log_2_CAM-MIC and Log_2_AZM-MIC. Panel A shows the differences according to species (*M. avium* or *M. intracellulare*), Panel B shows differences according to the presence of the *rrl* mutation (wild type or mutant), and Panel C shows differences according to a history of long-term administration of macrolides. Mann–Whitney *U*-test was used for the statistical analysis. In Panel B, for the mutant group, because the MICs of most strains were >64 µg/mL, no box or error bars are shown.

The Log_2_AZM-MIC was distributed slightly lower for *M. intracellulare* than for *M. avium*, whereas no statistically significant differences were observed in the Log_2_CAM-MIC according to species (Fig. 3A). The Log_2_AZM-MIC and Log_2_CAM-MIC were generally higher in isolates with *rrl* mutations than in wild-type isolates (Fig. 3B); however, four wild-type isolates had a CAM MIC of 16 µg/mL, and seven wild-type isolates had a CAM MIC of 32 µg/mL or higher. Isolates from patients with a history of macrolide antibiotic treatment had Log_2_AZM-MIC and Log_2_CAM-MIC compared to those from patients without a history of macrolide administration (Fig. 3C).

### Azithromycin MICs and azithromycin treatment outcomes

Among patients without a history of antimicrobial therapy for nontuberculous mycobacterial infection or prolonged macrolide monotherapy at the time of sample collection, only 18 patients were subsequently initiated on AZM-containing antimicrobial regimens to treat MAC infection. The details of these 18 patients are shown in Table 3. The predominant clinical subtype was nodular bronchiectasis. Consequently, treatment approaches often involved a combination of AZM and ethambutol, consistent with previous studies (11, 12).

**TABLE 3:** Clinical outcomes of azithromycin treatment^*h*^

Age	Sex	Type	Cavity	Species	*rrl* mutation	AFB smear	CAM MIC	AZM MIC	Time to treatment initiation (days)^*a*^	Treatment regimen^*b*^	Observation period (days)^*c*^	Culture-negative conversion^*d*^	Observation period after negative conversion (days)^*e*^	Bacteriologic relapse^*f*^
74	F	NB	+	*M. avium*	WT	−	0.5	8	42	AZM + EB + AMK (inh)	931	+	847	+
63	F	NB	−	*M. intracellulare*	WT	−	2	16	217	AZM + EB	223	−		
63	F	NB	−	*M. avium*	WT	−	2	32	550	AZM + EB	364	−		
82	M	NB	−	*M. intracellulare*	WT	−	0.25	8	418	AZM + EB	547	−		
53	F	NB	−	*M. avium*	WT	−	0.25	8	106	AZM + EB	811	+	790	−
63	F	NB	−	*M. avium*	WT	1+	8	32	663	AZM + EB	309	+	217	−
79	M	NB	−	*M. intracellulare*	WT	2+	0.125	4	26	AZM + EB	931	+	378	−
74	M	FC	*+*	*M. avium*	WT	−	2	8	58	AZM + EB + RFP	842	+	655	−
53	M	NB	−	*M. intracellulare*	WT	1+	0.5	16	353	AZM + EB + RFP	560	+	494	−
68	F	NB	−	*M. avium*	WT	±	4	64	376	AZM + EB	357	+	263	−
71	F	NB	−	*M. avium*	WT	−	0.5	16	223	AZM + EB	511	+	168	−
64	F	NB	−	*M. avium*	WT	−	0.5	8	445	AZM + EB	273	+	231	−
74	M	NB	−	*M. avium*	WT	−	2	32	63	AZM + EB	663	+	642	−
46	M	NB	−	*M. avium*	WT	−	2	32	313	AZM + EB	350	+	195	−
70	F	NB	−	*M. avium*	WT	−	1	32	111	AZM + EB	570	+	504	−
76	F	NB	*+*	*M. avium*	WT	−	1	8	51	AZM + EB + RFP	42	Indeterminate^*g*^		
76	F	NB	−	*M. avium*	WT	−	0.5	32	179	AZM + EB + RFP	763	Indeterminate^*g*^		
69	F	NB	−	*M. avium*	WT	−	2	32	50	AZM + EB	794	Indeterminate^*g*^		

^*a*^ Time to treatment initiation: Days from sample collection to initiation of antimicrobial therapy for MAC.

^*b*^ Treatment regimen: Antimicrobial regimen used for MAC treatment.

^*c*^ Observation period: Time (days) from antimicrobial therapy initiation to the last clinic visit, changing the antimicrobial therapy regimen, or loss to follow-up (due to transfer to another hospital or death).

^*d*^ Culture-negative conversion: Mycobacterial culture results of three serial sputum samples (collected at least 1 month apart) were negative.

^*e*^ Observation period after negative conversion: Time (days) from first negative samples collected to the last clinic visit, changing the antimicrobial therapy regimen, or loss to follow-up (due to transfer to another hospital or death).

^*f*^ Bacteriological relapse: multiple instances of mycobacterial culture positivity were confirmed after achieving culture-negative conversion.

^*g*^ Indeterminate: Three or more mycobacterial culture samples were not obtained after antimicrobial treatment was initiated.

^*h*^ AFB, acid-fast bacilli; AMK(inh), amikacin inhalation; AZM, azithromycin; EB, ethambutol; F, female; FC, fibrocavitary type; M, male; NB, nodular bronchiectasis; RFP, rifampicin; WT, wild type.

All 18 isolates were CAM susceptible, whereas AZM MICs were variable (ranging from 8 to 32 µg/mL). Additionally, no *rrl* mutations were observed. Three or more culture samples were collected from 15 patients after the initiation of AZM-containing regimens. Of these 15 patients, 11 achieved culture-negative conversion, and no bacteriological relapse was observed (microbiological cure), whereas four experienced treatment failure (persistence of positive cultures: *n* = 3; re-emergence of multiple positive cultures: *n* = 1). The means of the Log_2_AZM-MIC did not differ significantly between patients with microbiological cure and those with treatment failure (4.09 vs 3.75, *P* = 0.625).

## DISCUSSION

In this study, we investigated the susceptibility of 318 MAC strains to AZM over 1 year in a single institution in Japan, following the current CLSI recommendations, and found that the MIC values of AZM were correlated with those of CAM, but were consistently higher overall. Although the number of systematic studies on AZM MIC are limited, Zhang et al. (13) also reported that the AZM MICs were higher than the CAM MICs in a study using 32 *M*. *intracellulare* strains. Strains with *rrl* mutations associated with macrolide resistance had significantly higher AZM MICs than wild-type strains, suggesting that the MIC accurately reflected susceptibility to macrolides. The AZM MIC has also been reported to be higher than the CAM MIC in rapidly growing mycobacteria (14, 15). Although further validation is required for other mycobacterial species, the observed differences in the MICs of AZM and CAM may also be a characteristic common to the *Mycobacterium* genus.

The peak plasma concentration (Cmax) of AZM is generally reported to be 0.21–0.54 µg/mL following oral administration of 500 mg (16). In studies with of patients with MAC, a daily dose of 250 mg resulted in a Cmax of 0.24 µg/mL, whereas a thrice-weekly dose of 500 mg led to a Cmax of 0.65 µg/mL (17). Although these values are considerably below the MICs measured in our study, the high concentrations of AZM within alveolar macrophages, reported as 194 µg/mL after a 500-mg dose (18), suggest that a therapeutic effect can be achieved even at relatively high MICs. However, after 5 days of AZM administration (first day: 500 mg; second to fifth day: 250 mg daily), the plasma concentration was lower than the plasma concentration of CAM after 5 days of administration (first to fifth day: 500 mg twice daily). Therefore, although AZM showed better penetration into lung tissue and alveolar macrophages compared with CAM, CAM is reported to achieve higher final concentrations in the pleural fluid and alveolar macrophages of the lungs than AZM (19, 20). Current guidelines recommend a low dose of AZM (250 mg/day) (2), suggesting that the lung concentrations in patients receiving AZM may be lower than those in patients receiving CAM. Generally, the effectiveness of macrolide antibiotics is associated with the area under curve (AUC)/MIC ratio (21). However, with the currently recommended dosing regimens (2), it is possible that the AUC of AZM is lower than that of CAM. Conversely, considering that the MIC of AZM was higher than that of CAM, it is uncertain whether the clinical effectiveness of AZM is equivalent to that of CAM, particularly in cases with high AZM MICs. From this perspective, the validity of the current practice of estimating the effectiveness of AZM from the susceptibility testing results of CAM should be verified. Therefore, in the future, widespread implementation of AZM MIC measurements and extensive discussion on the correlation between AZM MIC and clinical effectiveness is necessary.

In our study, the patients with a history of macrolide administration, excluding those with short-term use, had higher AZM MICs. This is consistent with previous reports indicating a link between macrolide resistance and a history of macrolide administration, especially monotherapy (4). To prevent the emergence of macrolide resistance in MAC, more appropriate and prolonged administration of macrolides is warranted.

Among the patients without a treatment history included in this study, only 18 initiated AZM treatment after sample collection. Although no clear correlation was observed between the AZM MIC and the effectiveness of treatment, the limited sample size precludes definitive conclusions. Notably, microbial cure was achieved even in cases with AZM MIC values of 32 and 64 µg/mL. Considering this, using a breakpoint for AZM similar to the breakpoint of 16 µg/mL for CAM may be inadequate, and a higher breakpoint may be necessary.

This study has two main limitations. First, the upper limit of the measured range for AZM MIC was 64 µg/mL. Given that the AZM MIC values were generally higher than the CAM MIC values, concentrations exceeding 64 µg/mL should have been included in the measurement range. Second, this was a single-center study in which almost half of the patients had a history of macrolide treatment. Therefore, regional resistance patterns and the impact of macrolide treatment history might have influenced the results. Further larger multicenter studies evaluating AZM drug susceptibility are warranted.

In conclusion, the AZM MIC values were consistently higher than the CAM MIC values, suggesting the necessity of measuring AZM MIC to evaluate AZM susceptibility.

## Data Availability

The data sets generated and/or analyzed during the current study are available from the corresponding author on reasonable request.
